# Bilocular Uterus

**Published:** 1844-01-27

**Authors:** 


### BILOCULAR UTERUS.

A case of arrest of development is said to have occurred in Vienna, producing cleft vagina and bilocular uterus. The subject of it was a woman, thirty years of age, and who, being pregnant, applied to become an inmate at a lying-in charity. Externally she was well-shaped, and appeared robust; but on making examination, the vagina, at the depth of about two inches, was found divided into a double passage by a dense fibrous septum stretching across it. The posterior chamber was penetrable by the finger for about an inch and a half higher, when it was found to end in a small blind sac. The anterior passage of the vagina was so long that the os uteri could not be reached by the finger; the fœtus accordingly lay very high in the pelvis. The birth was at first lingering, but in the progress of the labour the septum in the vagina spontaneously ruptured, with little loss of blood; the liquor amnii was immediately discharged, and in a short time afterwards a living child was safely expelled. The mother, however, died of peritonitis four days afterwards, and on opening the body the cavity of the uterus, as far as the os internum, was seen to be separated into two chambers by a vertical septum. The fœtus had lodged in the left of these divisions, but the right cavity had also been dilated and lined with decidua during the pregnancy.—*London Lancet, from Oest. Med. Wochensch.*, Sept. 9, 1843.

